# Overall mortality for community‐dwelling adults over 50 years at risk of malnutrition

**DOI:** 10.1002/jcsm.13585

**Published:** 2024-08-29

**Authors:** Matthew Gittins, Nada AlMohaisen, Chris Todd, Simon Lal, Sorrel Burden

**Affiliations:** ^1^ School of Health Sciences University of Manchester Manchester UK; ^2^ Manchester Academic Health Science Centre Manchester UK; ^3^ Manchester University Foundation NHS Trust Manchester UK; ^4^ Salford Royal Foundation NHS Trust Salford UK

**Keywords:** Malnutrition, Mortality, Older people middle‐aged, Survival, Undernourished

## Abstract

**Background:**

It is well reported that malnutrition in acute care is associated with poorer health outcomes including increased mortality. However, the consequences of malnutrition on survival in community settings is uncertain. Malnutrition in people 65 years or over is often cited. Nevertheless, this study includes both middle‐aged and older adults as current public health policy is highlighting the need to increase disease‐free life years and is moving away from just extending life to increase overall longevity. The aim of this study is to describe the association of the risk of malnutrition using the Malnutrition Universal Screening Tool (MUST) with mortality in community‐dwelling middle‐aged and older adults.

**Methods:**

We used the UK Biobank to investigate the association between those at risk of malnutrition and mortality in participants aged ≥50 years. MUST identified risk of malnutrition and linked data to national death registries confirmed mortality. Years of life lost (YLL) and Cox proportional hazard models with hazard ratios (HR) and confidence intervals (CI) described risk associated with all‐cause mortality.

**Results:**

There were 502 408 participants recruited, 117 830 were ≤50 years leaving 384 578 eligible participants. Based on MUST scores 63 495 (16.5%) were at risk of malnutrition with 401 missing some data and excluded. Incidence of mortality for at risk participants was 755 per 100 000 person‐years, corresponding to 153 476 YLL. Of those at risk of malnutrition, 9.5% died versus 7.8% at low risk. Initial survival analysis reported an increased risk of mortality (HR 1.29, 95% CI: 1.25 to 1.33) that decreased after adjusting for confounders (HR 1.14, 95% CI: 1.11 to 1.18) in those at risk of malnutrition versus those at low risk.

**Conclusions:**

Risk of malnutrition was associated with increased overall mortality. Modest effect sizes are demonstrated but are supportive of public health policies, which advocate wide‐scale community, based nutritional screening for middle‐aged and older adults.

## Introduction

Malnutrition can be defined as ‘deficiencies, excesses, or imbalances in a person's intake of energy and/or nutrients’.^1^ In this study, we focus on undernutrition only and use the term malnutrition. Malnutrition in adults is categorised into three groups: inflammatory disease‐related; non‐inflammatory disease‐related, and non‐disease related malnutrition.^2^ Disease states often associated with malnutrition and inflammation include cardiovascular, respiratory, gastrointestinal, musculoskeletal diseases, infections, and cancer.^3^ Non‐inflammatory malnutrition can occur where physiological mechanisms do not lead directly to malnutrition, but where other disease processes and symptoms result in reduced nutritional intake, such as some psychiatric disorders, neurological disorders, or cognitive impairment.^4^ Malnutrition not related to disease refers to hunger or issues within society including the consequences of deprivation and poverty, social isolation, and food insecurity.^4^

Recommendations for identifying malnutrition initially suggest starting with screening using a validated tool compliant with the Global Leadership Initiative on Malnutrition (GLIM).^5^ Many hospitals in the UK use the Malnutrition Universal Screening Tool (MUST),^6^ and other tools used internationally include the Mini‐nutritional Assessment Tool, Geriatric Nutrition Risk Index, and the Nutrition Screening Tool.^4^ Data from UK national hospital screening estimates that the prevalence of malnutrition is 29% in all adults.^7^ Similarly, European data from over 20 000 participants in 25 countries estimated that nearly a third of people were at risk of malnutrition in hospital settings.^8^ Screening for risk of malnutrition in hospitals is now well established internationally, with consistent evidence being generated in low, medium, and high‐income countries.^9^ It is worth noting MUST use for community screening only includes two questions (percentage weight loss and body mass index [BMI]), ‘acute disease effect’, which is the third question is not applicable in the community setting.^6^ To differentiate between the underlying causes of malnutrition nutrition screening tools are inadequate, and a fuller nutritional assessment is required including criteria specified by GLIM. Screening tools can identify risk of malnutrition whereas a fuller nutritional assessment can diagnose malnutrition, establish underlying causes, and inform a treatment plan.^10^

Unintentional weight loss is a predictor of poor outcomes in acute illness and is one of the five components of frailty in the phenotype model.^11^ People who are at risk of malnutrition or malnourished have prolonged recovery from illness and incur substantially more complications after treatment.^12^ In addition, observational studies have shown people who are at risk of malnutrition or malnourished also have poorer outcomes after surgery compared with their well‐nourished counterparts, and increased utilisation of healthcare resources.^13^, ^14^ However, attenuating risk of malnutrition with nutritional interventions in acute settings with oral nutritional supplements has been shown to reduce hospital length of stay, complications, improve functionality, and quality of life.^15^, ^16^ Interventions provided to older people in the community have also shown benefits in reducing infections, pressure ulcer wounds, and improving healing of fractures.^17^

Risk of malnutrition assessed using the Mini Nutritional Assessment instrument is associated with increased rates of mortality in older adults in hospitals regardless of the cause of death.^18^ Numerous studies in different disease states including chronic obstructive pulmonary disease, heart failure, and cancer, have reported substantial differences in outcomes between people who are at risk of malnutrition and those who are well‐nourished or at low risk measured using a number of different tools (Nutritional Screening Tool, Subjective Global Assessment, Mini‐Nutritional Assessment, and MUST).^19^

Internationally, people over the age of 60 are projected to nearly double from 1.4 billion in 2019 to 2.1 billion by 2050, with one in six people in 2060 being over the age of 60 years [S1]. With an ageing population, public health policy is now not only aiming to improve longevity but also to increase disease‐free life years by promoting healthy lifestyles and preventative healthcare measures [S2]. The healthy lifespan gap is approximately 9.2 years, calculated from the health‐adjusted life expectancy and total lifespan [S3]. Interventions that improve healthy life years lived without chronic diseases are therefore aligned to current public health strategy and can include regular screening programmes, physical activity promotion, and nutritional interventions.^20^ Preventing morbidity and mortality in older adults needs to begin earlier in the life, as reiterated by the World Health Organisation in their decade of healthy ageing, which targets high, middle, and low‐income countries. [S1] Malnutrition and mortality is therefore of interest in people aged 50 years or over, as identifying and treating malnutrition has been shown to decrease frailty, falls, and retain functionality while promoting independent living.^21^

In community‐dwelling middle‐aged and older people aged ≥50, the overall prevalence rate for individuals at risk of malnutrition has been reported to be 17% (95% confidence intervals [CI]: 0.01 to 0.46) based on five studies, including 4213 participants.^22^ The wide range of precision is due to the inclusion of different populations and the use of a variety of validated and modified screening tools, indicating more robust evidence is required. For community‐based settings, including residential and nursing homes, outpatients and health centres, the prevalence of individuals at risk of malnutrition was noted as 26.5% (95% CI: 22.4 to 32.7).^23^ However, there remains a poor uptake of nutritional screening and identification of malnutrition in community settings. Malnutrition identified in community settings is associated with an increased risk of falling, loss of independence, decreased mobility and functionality^21^ with an indisputable overlap with frailty and sarcopenia.^24^, ^25^ However, there is a lack of data on any association with overall mortality in people who are at risk of malnutrition or malnourished in large community‐dwelling populations who are middle‐aged or over. A James Lind Alliance Priority Setting Partnership identified malnutrition in community settings as one of the top 10 research priorities.^26^

To allow a better understanding of the risk of malnutrition in community‐dwelling adults, we investigated the relationship between risk of malnutrition, all‐cause mortality and overall survival for people who are 50 years and over. Our core aim is to describe the risk associated with death due to risk of malnutrition and the corresponding years of life lost (YLL).

## Methods

We used data from the UK Biobank, a national level prospective cohort study that selected eligible people who were registered in the National Health Service. Detailed methods for the UK Biobank have been previously reported.^27^ Half a million participants aged from 37 to 70 years were voluntarily recruited (with a 5.5% response rate) at 22 assessment centres across England, Scotland, and Wales between February 2006 and 2010.^28^ With participant consent, data were collected directly through questionnaires, physical measurements, imaging, blood, saliva, and urine tests. Health and mortality data were obtained through linkage to health records and death registries. Participants were included in this study if they were aged 50 years or older at their baseline assessment so participants could be followed up within the cohort as they aged up to the final censored point. Data for males and females were based on biological sex.

Participants were identified as being at risk of malnutrition if they met the criteria proposed by MUST using BMI and the weight loss variable. The acute disease effect score is not applicable to community‐dwelling individuals.^6^ Here participants were defined as at risk of malnutrition if they report weight loss in the previous 12 months or they were considered to have a low BMI < 22 kg/m^2^ if 70 years old or above, or BMI < 20 kg/m^2^ if under 70. The UK Biobank contains self‐reported information only on whether participants lost weight during the preceding year, unplanned or otherwise, however, recall weight has been used previously for the percentage weight loss variable in MUST.^29^ The MUST score only includes two categories low risk and medium combined with high risk of malnutrition as the weight loss variable did not detail the amount of weight loss to calculate percentage weight loss. For BMI height and weight were measured at baseline by a research nurse at one of the centres used by the UK Biobank for data collection. Height was measured using a Seca 202 height‐measuring rod and weight was measured using Tanita BC 418 MA body composition analyser.^30^

Using the linked health data, we identified participants who were reported to have died of any cause during the follow‐up period until 30 April 2022, which was the censoring date. The maximum follow‐up duration for participants in this study was 16 years. We also identified if a participant had developed any cancer prior to baseline assessment at any point during their life or follow‐up period. For health‐related data, a self‐reported variable was used from a question asked at baseline by a trained nurse.

Written consent was obtained from all participants for baseline assessment and linkage to registries and the UK Biobank obtained ethical approval from the National Health Service Research Ethics Service (11/NW/0382). This study was registered with the UK Biobank (No. 49810). The full study protocol^31^ is available, although this report is only presenting the risk of malnutrition data.

### Statistical analysis

Standard descriptive statistics for time‐to‐event data describe the rates of mortality within the sample and its associated impact on life expectancy. This includes the corresponding incidence rates, Kaplan–Meier plots, and YLL for those at risk of malnutrition. YLL was calculated for age and gender separately based on UK life‐expectancy tables calculated by the Office of National Statistics [S3]. Total YLL was converted into a rate per 100, 000 persons at risk of malnutrition. Survival analysis then estimated the risk of all‐cause mortality associated with being at risk of malnutrition. The primary outcome, time to death, was right censored. To facilitate the inclusion of left truncation into the analysis, time was calculated based on age of participant at the baseline assessment until age of death or censoring. Hazard ratios (HR) and 95% CI, associated with risk of mortality, were then produced for participant at risk of malnutrition versus low risk using Cox proportional HR with increasing adjustment for known and measured confounders. The assumption of proportionality was checked prior to running the HR and data were assessed as adhering to this assumption.

The initial ‘unadjusted’ model contained risk of malnutrition alone (and age as part of the analysis time). Additional covariates were included in two stages, first the time‐invariant factors: gender, ethnicity, qualification, and deprivation (in the form of the Townsend score), and co‐morbidities including hypertension, diabetes, ischaemic heart disease, osteoporosis, Parkinson's, thyroid disease, cerebrovascular disease, chronic kidney disease, atrial fibrillation, anaemia, and respiratory disease. The medical conditions included were noted as the cause of death reported on the death certificate documented in the UK Biobank using the International Classification of Disease (ICD) code. Second, a time‐varying covariate accounting for whether or not the participant developed cancer before or during follow‐up was included. Additional analyses included an interaction effect between risk of malnutrition and gender, to investigate whether risk of malnutrition was differentially associated with mortality through gender. Due to the considerable influence cancer has on both malnutrition status through weight loss and risk of mortality, a final analysis was performed restricted to those reportedly cancer‐free at baseline.

We performed a sensitivity analysis to explore the influence of recruitment centre. Participants recruited at the same centre may share similar characteristics including underlying hazard function that differed to other centres due to being sourced from a similar underlying population. In the context of a survival analysis, this bias maybe accounted for, in a shared frailty model. To understand whether shared frailty by recruitment centre was an important factor under this assumption, we have re‐fitted the analysis using a shared frailty model. Population Attributable Fraction (PAF) is an epidemiological measure used to assess the impact of an exposure in a population and was calculated using observed (number of cases) minus expected (number of cases under no exposure) divided by observed [S4].

## Results

From 9 238 453 invited participants 502 408 people were initially recruited to the UK Biobank who had not requested withdrawal by September 2022. At study entry, 117 830 were aged under 50 years leaving 384 578 participants aged 50 to 73 eligible to be included. Based on the MUST criteria 63 495 (16.5%) were deemed at risk of malnutrition with 401 missing information at baseline on both weight change and BMI and so were dropped from the formal survival analysis. Of those at risk of malnutrition 9.5% died during follow‐up, compared with 7.8% in the low‐risk comparison group. The average age of included participants was 60 (SD 5.4) years with a median age of 61 (IQR 56–64) years at baseline assessment. Fifty‐four per cent of the sample were female, 95% were white British, and 53% had one or more co‐morbidities recorded. Fourteen per cent of the sample had a cancer diagnosis recorded at baseline. Table 1 provides a detailed breakdown of the sample split by whether the participant was at risk of malnutrition or had died by April 2022. Table 2 shows co‐morbidities for people who died who and were at risk of malnutrition.

**Table 1:** Participant characteristics for people at risk of malnutrition and those who died

Characteristics		At risk of malnutrition	Died	Total
MUST	Not	‐	25 088 (7.8)	320 692
	At risk	‐	6056 (9.5)	63 485
	Missing	‐	58 (14.5)	401
BMI score	>20.0	55 099 (14.7)	29 891 (8)	374 165
	18.5–20.0	6159 (100)	583 (9.5)	6159
	<18.5	1913 (100)	292 (15.3)	1913
	Missing	314 (13.4)	436 (18.6)	2341
Weight change	Prefer not to say	8 (2.9)	40 (14.7)	272
	Do not know	174 (2.7)	621 (9.8)	6363
	No ‐ weigh about the	6037 (2.8)	16 795 (7.8)	216 222
	Yes ‐ gained weight	365 (0.4)	8216 (7.9)	104 177
	Yes ‐ lost weight	56 891 (100)	5461 (9.6)	56 891
	Missing	10 (1.5)	69 (10.6)	653
Sex	Female	36 960 (17.7)	12 567 (6)	208 683
	Male	26 525 (15.1)	18 635 (10.6)	175 895
Ethnicity	White	60 378 (16.5)	30 015 (8.2)	366 777
	Mixed	269 (17.1)	103 (6.5)	1574
	Asian or Asian British	970 (15.8)	394 (6.4)	6134
	Black or Black British	849 (19.3)	269 (6.1)	4391
	Chinese	193 (20.5)	42 (4.5)	942
	Other	565 (21)	158 (5.9)	2689
	Missing	261 (12.6)	221 (10.7)	2071
Smoking	Never	32 679 (16.2)	11 646 (5.7)	201 670
	Ever	23 592 (16.4)	13 409 (9.3)	144 058
	Current	6911 (18.9)	5853 (16.0)	36 524
Qualification	College or university	19 302 (16.7)	6929 (6)	115 297
	A levels/AS levels	6876 (17.4)	2634 (6.7)	39 491
	O levels/GCSEs or equiv	12 869 (16.5)	5666 (7.3)	77 982
	CSEs or equivalent	2549 (16.7)	922 (6.1)	15 238
	NVQ or HND or HNC	4374 (16.3)	2472 (9.2)	26 834
	Other professional qualification	3788 (16.6)	1847 (8.1)	22 833
	None of the above	12 553 (15.9)	9819 (12.4)	79 159
	Prefer not to say	718 (16.4)	497 (11.3)	4383
	Missing	456 (13.6)	416 (12.4)	3361
**Total**		**63 485 (16.5)**	**31 202 (8.1)**	**384 578**

BMI, body mass index; CSE, Certificate of Secondary Education; GCSE, General Certificate Secondary Education; HNC, High National Certificate; HND, Higher National Diploma; MUST, Malnutrition Universal Screening Tool; NVQ, National Vocational Qualification.

**Table 2:** Co‐morbidities for participants at risk of malnutrition and for those who died

Co‐morbidities		At risk of malnutrition	Died	Total
Hypertension	None	40 197 (15.8)	14 807 (5.8)	253 815
	Present	23 288 (17.8)	16 395 (12.5)	130 763
Diabetes	No	56 900 (15.8)	26 795 (7.5)	359 408
	Yes	6386 (27.4)	4192 (18)	23 318
	Prefer not to say	160 (17.3)	111 (12)	925
	Do not know	28 (10.5)	35 (13.2)	266
	Missing response	11 (1.7)	69 (10.4)	661
Ischaemic heart disease	None	54 326 (16.3)	22 872 (6.8)	333 902
	Present	9159 (18.1)	8330 (16.4)	50 676
Osteoporosis	None	59 996 (16.3)	28 711 (7.8)	367 900
	Present	3489 (20.9)	2491 (14.9)	16 678
Parkinson's	None	63 066 (16.5)	30 275 (7.9)	382 407
	Present	419 (19.3)	927 (42.7)	2171
Thyroid disease	None	63 341 (16.5)	31 125 (8.1)	383 810
	Present	144 (18.8)	77 (10)	768
Cerebrovascular	None	60 189 (16.4)	26 293 (7.2)	366 397
	Present	3296 (18.1)	4909 (27)	18 181
Kidney disease	None	60 241 (16.4)	27 273 (7.4)	367 568
	Present	3244 (19.1)	3929 (23.1)	17 010
Atrial fibrillation	None	57 708 (16.4)	24 601 (7)	352 747
	Present	5777 (18.1)	6601 (20.7)	31 831
Arthritis	None	62 498 (16.5)	30 389 (8)	379 798
	Present	987 (20.6)	813 (17)	4780
Anaemia and haematinic deficiency	None	60 213 (16.4)	28 327 (7.7)	367 706
	Present	3272 (19.4)	2875 (17)	16 872
Respiratory disease	None	45 768 (15.9)	11 607 (4)	288 324
	Present	17 717 (18.4)	19 595 (20.4)	96 254
Cancer	None	59 901 (16.5)	27 466 (7.6)	363 973
	Malignancy	3584 (17.4)	3736 (18.1)	20 641
**Total**		**63 485 (16.5)**	**31 202 (8.1)**	**384 578**

**Figure 1:**
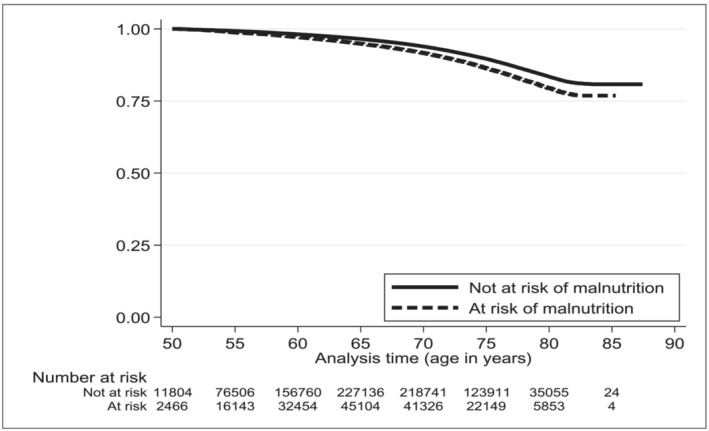
Kaplan–Meier plot comparing participants at risk of malnutrition versus low risk of malnutrition.

Figure 1 further describes the distribution of time to death using Kaplan–Meier plots within the sample during follow‐up time, where follow‐up time is based on participant's age. Figure 1 reports the time‐to‐death in terms of risk of malnutrition versus low risk, and Figure S1 further splits by gender. Tables 3 and 4 report incidence rates per 100 000 person‐years (pys) and YLL per 100 000 persons by age at death and gender for the whole sample and for those without cancer at baseline, respectively. For all participants, the overall incidence rate (IR) was 755 (95% CI: 736 to 774) per 100 000 pys for those aged over 50 with malnutrition, for males and females respectively this was 1022 (95% CI: 988 to 1057) and 569 (95% CI: 548 to 591) per 100 000 pys. This corresponded to 190 492 and 126 913 YLL per 100 000 persons at risk of malnutrition for men and women respectively (see Figure S2) and 153 476 for any 100 000 persons at risk of malnutrition. In those free from cancer at baseline entry (Table 4), the overall IR dropped to 689 (95% CI: 671 to 708) per 100 000 pys for those aged over 50 years at risk of malnutrition, and 939 (95% CI: 905 to 973) and 514 (95% CI: 493 to 535) for males and females respectively. The corresponding YLL was 174 473 and 113 759 for 100 000 at risk of malnutrition men and women respectively, and 139 232 for any 100 000 at risk of malnutrition (Figure S2).

**Table 3:** Incidence rates per 100 000 person years and years of life lost per 100 000 persons by age and gender for all participants at risk of malnutrition

Age interval	Alive at entry	Deaths	Person‐time	Rate per 100 000 pys	95% CI	YLL	YLL per 100 000 ps
50‐	63 485	118	39 339	300	250, 359	3422	5390
55‐	63 367	387	111 327	348	315, 384	9755	15 394
60‐	62 980	933	194 502	480	450, 512	19 790	31 423
65‐	56 380	1514	217 800	696	661, 732	26 123	46 334
70‐	41 328	1885	162 583	1159	1108, 1213	25 736	62 273
75‐	22 149	1095	67 487	1621	1528, 1720	11 572	52 246
80‐	5853	124	8953	1385	1162, 1652	1037	17 717
85‐	4	0	0	0	‐	‐	‐
Total	63 485	6056	801 991	755	736, 774	97 434	153 476

Female only							
50‐	36 960	50	23 828	210	159, 277	1541	4169
55‐	36 910	180	67 399	267	231, 309	4808	13 026
60‐	36 730	432	116 603	371	337, 407	9827	26 755
65‐	32 873	676	127 379	532	493, 573	12 596	38 317
70‐	23 902	805	93 593	860	803, 922	12 000	50 205
75‐	12 669	491	38 503	1273	1165, 1390	5642	44 534
80‐	3363	54	5181	1042	798, 1361	493	14 660
85‐	1	0	0	0	‐	‐	‐
Total	36 960	2688	472 485	569	548, 591	46 907	126 913

Male only							
50‐	26 525	68	15 511	438	346, 556	1881	7091
55‐	26 457	207	43 928	471	411, 540	4946	18 694
60‐	26 250	501	77 899	643	589, 702	9963	37 954
65‐	23 507	838	90 421	927	866, 992	13 527	57 545
70‐	17 426	1080	68 991	1565	1475, 1662	13 737	78 830
75‐	9480	604	28 983	2084	1924, 2257	5930	62 553
80‐	2490	70	3772	1856	1468, 2346	544	21 847
85‐	3	0	0	0	‐	‐	‐
Total	26 525	3368	329 506	1022	988, 1057	50 528	190 492

ps, persons; pys, person years; YLL, years of life lost.

**Table 4:** Incidence rates per 100 000 person years and years of life lost per 100 000 persons by age and gender for all participants at risk of malnutrition and without cancer at baseline assessment

Age interval	Alive at entry	Deaths	Person‐time	Rate per 100 000 pys	95% CL	YLL	YLL per 100 000 ps
50‐	59 901	87	37 821	230	186, 284	2523	4212
55‐	59 814	308	106 881	288	258, 322	7748	12 954
60‐	59 506	788	185 716	424	396, 455	16 710	28 080
65‐	53 220	1298	206 052	630	597, 666	22 372	42 036
70‐	38 894	1675	152 693	1096	1045, 1150	22 840	58 724
75‐	20 719	973	62 955	1546	1451, 1646	10 272	49 578
80‐	5420	112	8235	1360	1130, 1637	937	17 284
85‐	3	0	0	0		0	0
Total	59 901	5241	760 353	689	671, 708	83 401	139 232

Female only							
50‐	34 769	37	22 801	162	118, 224	1140	3278
55‐	34 732	134	64 364	208	176, 247	3584	10 319
60‐	34 598	360	110 737	325	293, 361	8202	23 706
65‐	30 934	570	119 999	476	438, 517	10 628	34 358
70‐	22 420	714	87 637	814	756, 876	10 626	47 396
75‐	11 827	429	35 833	1197	1089, 1316	4934	41 717
80‐	3112	48	4760	1008	760, 1338	439	14 098
85‐	1	0	0	0		0	0
Total	34 769	2292	446 132	514	493, 535	39 553	113 759

Male only							
50‐	25 132	50	15 020	333	252, 439	1383	5504
55‐	25 082	174	42 517	409	353, 475	4164	16 603
60‐	24 908	428	74 978	571	519, 628	8508	34 156
65‐	22 286	728	86 052	846	787, 910	11 743	52 694
70‐	16 474	961	65 056	1477	1387, 1574	12 214	74 140
75‐	8892	544	27 122	2006	1844, 2182	5338	60 034
80‐	2308	64	3475	1842	1442, 2353	498	21 581
85‐	2	0	0	0		0	0
Total	25 132	2949	314 221	939	905, 973	43 849	174 473

ps, persons; pys, person years; YLL, years of life lost.

**Figure 2:**
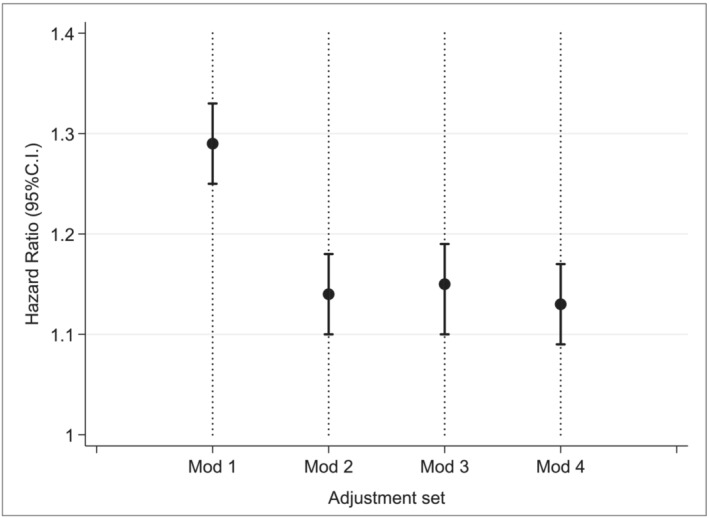
Hazard ratios associated with ‘at risk of malnutrition’ versus ‘low risk’ under four modelling conditions of increasing confounder adjustment. Mod1 = no adjustment, mod 2 = adjust for known fixed confounders: Gender, ethnicity, qualification, and deprivation (in the form of the Townsend score), and co‐morbidities hypertension, diabetes, ischaemic heart disease, osteoporosis, Parkinson's, thyroid disease, cerebrovascular disease, chronic kidney disease, atrial fibrillation, anaemia, and respiratory disease, smoking status, baseline cancer presence. Mod 3 = plus adjustment for time‐varying cancer occurrence mod 4 = restricted sample of healthy participants without cancer.

Survival analysis in Table 5 and Figure 2 reported HR and 95% CI for those at risk of malnutrition versus low risk as the reference category. The four models of increasingly restrictive adjustment sets are reported along with results of the investigation of interaction between gender and malnutrition status. The full results for model 3 with all covariates including time‐varying cancer presence can be found in Table S1. Once confounding was applied results indicated the risk of mortality increased by 13% (HR 1.13 95% CI: 1.10 to 1.17) in those at risk of malnutrition. The interaction model found no evidence to suggest an interaction between males and females was present. Analysis restricting the data to low‐risk participants without cancer at baseline resulted in very little change in risk relating to 13% (95% CI: 10 to 18). Sensitivity analysis (Table S1) incorporated shared frailty by recruitment centre within the survival modelling and was found to have no effect on the observed effects in the model.

**Table 5:** Hazard ratio and 95% confidence intervals from survival analysis comparing participants at risk of malnutrition versus low risk of malnutrition

Risk of malnutrition (MUST)	Hazard ratio	95% CI
No adjustment (Mod 1)		
Low risk		
At risk of malnutrition	1.29	1.25, 133
Adjusted for baseline confounders (Mod 2)		
Low risk		
At risk of malnutrition	1.14	1.11, 1.18
Adjusted for time‐varying cancer presence (Mod 3)		
Low risk		
At risk of malnutrition	1.15	1.11, 1.19
Interaction model with gender		
Low risk		
At risk of malnutrition	1.14	1.08, 1.20
Female		
Male	1.49	1.45, 1.54
MUST*Gender interaction	1.02	0.95, 1.09
Restricted to those without cancer at baseline only (Mod 4)		
Low risk		
At risk of malnutrition	1.13	1.09, 1.17

Mod 1 = No adjustment. Mod 2 = adjust for known fixed confounders: gender, ethnicity, qualification, and deprivation (in the form of the Townsend Score), and co‐morbidities including hypertension, diabetes, ischaemic heart disease, osteoporosis, Parkinson's, thyroid disease, cerebrovascular disease, chronic kidney disease, atrial fibrillation, anaemia, and respiratory disease, smoking status, baseline cancer presence. Mod 3 = plus adjustment for time‐varying cancer occurrence. Mod 4 = restricted sample of healthy participants without cancer.

MUST, Malnutrition Universal Screening Tool.

From the HR reported above we calculated a Population Attributable Fraction (PAF), which is the percentage of deaths attributable to being at risk of malnutrition. Using HR of 1.13 and its CIs and the prevalence of at risk of malnutrition in those that died (Table 1), the PAF was calculated to be 2.25% (1.73% to 2.83%). So approximately 2.3% of deaths in those over 50 years of age may be attributable to being at risk of malnutrition.

## Discussion

From the UK Biobank data and using MUST scores our data demonstrates that the risk of mortality is higher in people living in the community who were at risk of malnutrition using MUST compared with their low risk counterparts. Data show PAF for risk of malnutrition was 2.3%. This means that there are a substantial number of deaths attributable to risk of malnutrition in the UK each year. Given the prevalence of 16.5% of participants at risk of malnutrition in the UK Biobank (see results above) we estimate that there are 4.2 million people, 50 years or over who are at risk of malnutrition.[S3] This suggests that of those currently aged 50 or over, we would expect 94 000 (95% CI: 72 600 to 118 860) of them to die due to being at risk of malnutrition. Preventing risk of malnutrition may therefore delay death in approximately 100 000 individuals.

Risk of malnutrition has been frequently associated with morbidity and mortality, particularly in hospitalised individuals with different underlying diseases.^12^ Data exist on mortality and risk of malnutrition in a number of specific disease states and range from a HR of 1.32 reported for patients with neoplasm and a HR of 5.44 in patients with mental or behavioural illness for risk of malnutrition and those who were malnourished.^18^, ^21^ International data, indicate that the risk of malnutrition is associated with 30‐ day postoperative mortality across all income settings although one in three deaths in low and middle‐income countries were mediated by risk of malnutrition (OR 1.41, 1.22 to 1.64).^13^ Moreover, all‐cause mortality has been shown to be strongly associated with risk of malnutrition identified using the Mini Nutrition Assessment instrument in older adults in Sweden recruited while in hospital.^18^

An association between all‐cause mortality and malnutrition measured using some GLIM criteria in Hong‐Kong reported adjusted HR of 1.62 (95% CI: 1.39 to 1.89) in 3702 Chinese older people ≥65 years (mean 72.2, range 65–98) recruited from community centres.^32^ The HR reported was considerably higher than those from the UK Biobank data, which could be due to different risk profiles in the sample populations or methodological differences in recruiting study samples. Additionally, malnutrition was measured using objective measurements including muscle mass, BMI and inflammation, in contrast to the UK Biobank data that used self‐reported weight loss with BMI to obtain a MUST score.^32^

A prevalence rate of 4% for malnutrition was reported from the UK Biobank dataset including 142 880 participants 37 to 73 years old who had data that could be mapped to the GLIM criteria, which included at least one phenotypical criterion (low muscle mass or low BMI) and one aetiological criterion (anorexia or inflammation).^33^ However, in this study, the self‐reported weight loss variable was not included, which was influential in determining risk of malnutrition using MUST. The comparison of the UK Biobank data in determining malnutrition using the GLIM criteria and MUST score was 4% and 16.5%, respectively, highlighting the variance with criteria used to measure nutritional status. This is an example of the difference between risk of malnutrition used for screening and the identification of malnutrition using more of the measurements recommended by the GLIM criteria.^5^

Risk of malnutrition is a modifiable risk factor that, in the absence of inflammation, can be attenuated by the provision of nutritional support.^16^ Attenuation can be achieved by increasing the frequency of daily eating episodes, maximising the nutritional content of food or offering oral nutritional supplements.^4^ There is evidence demonstrating that addressing malnutrition in older people with specific diseases including cancer can lead to improved clinical outcomes, quality of life and functionality particularly in acute settings.^16^ For community dwelling older adults there is some evidence to suggest that addressing contributory factors such as social isolation, food insecurity, poverty and mental illness can lead to improved nutritional status.^17^ Modifiable risk factors for community dwelling older adults have been reviewed and include both social and clinical risks.^19^ Clinical risk factors are related to inflammation associated with many chronic diseases where even low‐grade inflammation has been demonstrated to inhibit muscle synthesis.^34^ Malnutrition coexists with both frailty and sarcopenia, where there are a number of overlapping components between all three conditions.^35^ Frailty is important, as it is associated with chronic conditions that increases mortality and morbidity [S5].

Given the adverse effects of malnutrition in older adults, international public health policies have been slow to respond to nutrition screening recommendations for community‐dwelling older adults. Barriers to nutritional screening implementation in primary healthcare include limited communication between healthcare settings, a lack of resources for completing screening, and a shortage of community dietitians. Currently, BMI is included in health assessments in primary care for adults over 40 years in the UK, although unintentional weight loss is not routinely recorded as part of the health check. Weight is recorded in a third of patients in primary care and was associated with either high or low BMI, co‐morbidities, deprivation, and number of consultations.^36^

One strength of the analyses presented here is the large sample of older adults who are community dwelling and where MUST scores can be mapped to mortality in participants recruited across England, Wales, and Scotland. Data used in this study were primarily collected at assessment centres so there is a low level of missingness within the dataset and data are valid and reliable. Data for mortality were from the national death registry, which is a reliable source for matching the mortality variable.

Limitation of this study relate to the use of participant recall to calculate MUST, although BMI used recorded height and weight. For the MUST variable, a recall question was used that asked if participants had lost any weight during the previous 12 months. Weight loss was therefore not quantified, which may have led to an overestimation of risk of malnutrition in this study. Other studies have used recall weight loss to calculate the percentage weight loss variable for MUST.^29^ The prevalence of older people who are community‐dwelling at risk of malnutrition or malnourished varies considerable within the evidence base. One study reported the risk of malnutrition as 35% using the Canadian screening tool,^37^ one studies using GLIM reported a prevalence of 23% in Spain in the SarcoPhAge study^38^ and 17% (95% CI: 1 to 46) from a systematic review including five studies that used the Mini Nutritional Assessment tool.^22^

In addition, data from the UK Biobank cannot distinguish between medium and high risk of malnutrition as the amount of weight loss was not recorded. There are three parts to MUST when the tool is used in acute settings and for community use, it is notes in the explanatory booklet that the acute disease effects is unlikely to occur in community settings.^6^ MUST is a screening tool not an assessment tool so our results relate to the risk of malnutrition which needs further assessment for a diagnosis of malnutrition to be confirmed with additional clinical data such as oral nutritional intake, muscle mass, inflammatory status, and functionality.

It is also important to note that the UK Biobank is not a representative sample of the UK population as there was only a response rate of 5.5% so participants recruited were more affluent, better educated and were predominantly white compared with the general population such that results may not be generalisable to the population.^28^ This is particularly important as people in lower socioeconomic groups face higher rates of food insecurity and are more likely to have malnutrition due to poverty.^39^, ^40^ In addition, the UK Biobank has limited data on people 80 years or over due to the age of participants at recruitment. In this study, we adjusted for all known confounders where there was a suitable variable, although it is possible that there is some residual confounding that is not accounted for that could potentially influence the results. For the incidence of mortality, YLL per 100 000 persons increases up to 65 years old for participants at risk of malnutrition then for those people 75 years or over this decreases. Albeit the rate of person years increases up to 75 years as this is standardised to 100, 000 person years. These figures reflect the sample recruited, as there were only 5857 (9.2%) participants 80 years or over recruited and only 22 149 (35%) participants recruited were 75–79 years that were at risk of malnutrition.

This study demonstrates that there is a 14% increase in mortality from the hazard ratios for participants over 50 years old compared with their low risk counterparts using the MUST. The data from the incidence of mortality used to calculate the YLL shows an overall increase in YLL up to 65 years in people who are at risk of malnutrition. This extends up to 75 years when the data is standardised and presented per 100 000 person years. However, in the age category for participants over 80 years caution is required in the interpretation due to the low numbers in the sample size. It is noteworthy that when the results from the Cox Proportionate Hazards models are applied at the population level, this risk amounts to a considerable health burden for community‐dwelling older adults and is particularly poignant as risk of malnutrition can be modifiable where there is an absence of an inflammatory response. These data support public health policies aimed at wide scale community based nutritional screening for middle‐aged and older adults including unintentional weight loss. If people at risk of malnutrition can be identified in the community, then treatment pathways can be established and implemented at an early stage to improve outcomes. Due to the limitations of the data on participants 75 years or older, more research on the consequences of malnutrition on mortality and YLL needs to be investigated in the older age categories.

## Conflict of interest

SL, CT, and MG declare no competing interests and SB has received education grants from Nutricia to attend a scientific conference in 2022 and 2023.

## Supporting information


**Data S1.** Kaplan Meier plot comparing MUST definition of malnutrition defining at risk vs not for males and female separately.
**Data S2.** Years of Life lost (per 100,000 persons) for those with malnutrition by age at death and gender.
**Data S3.** Years of Life lost (per 100,000 persons) for those with malnutrition by age at death and gender but did not have cancer at baseline.
**Data S4.** Sensitivity Analysis Hazard ratios and 95% confidence intervals for survival analysis (with shared frailty) comparing those at risk of malnutrition vs low risk of malnutrition.

## References

[1] World Health Organization . Malnutrition. https://www.who.int/news‐room/questions‐and‐answers/item/malnutrition. Accessed 12/10/2022.

[2] Cederholm T , Barazzoni R , Austin P , Ballmer P , Biolo G , Bischoff SC , et al. ESPEN guidelines on definitions and terminology of clinical nutrition. Clin Nutr 2017;36:49–64. 27642056 10.1016/j.clnu.2016.09.004

[3] Arends J , Baracos V , Bertz H , Bozzetti F , Calder PC , Deutz NEP , et al. ESPEN expert group recommendations for action against cancer‐related malnutrition. Clin Nutr 2017;36:1187–1196. 28689670 10.1016/j.clnu.2017.06.017

[4] Dent E , Wright ORL , Woo J , Hoogendijk EO . Malnutrition in older adults. Lancet 2023;401:951–966. 36716756 10.1016/S0140-6736(22)02612-5

[5] Cederholm T , Jensen GL , Correia M , Gonzalez MC , Fukushima R , Higashiguchi T , et al. GLIM criteria for the diagnosis of malnutrition ‐ a consensus report from the global clinical nutrition community. Clin Nutr 2019;38:1–9. 30181091 10.1016/j.clnu.2018.08.002

[6] Elia M. The 'MUST' Report BAPEN Redditch Worcs: 2003.

[7] Russell C , Elia M . Nutrition screening in hospitals in the UK, 2007–2011 In: BAPEN editor. Redditch: Worcs; 2014.

[8] Schindler K , Pernicka E , Laviano A , Howard P , Schütz T , Bauer P , et al. How nutritional risk is assessed and managed in European hospitals: a survey of 21,007 patients findings from the 2007‐2008 cross‐sectional nutritionDay survey. Clin Nutr 2010;29:552–559. 20434820 10.1016/j.clnu.2010.04.001

[9] Jones D , Knight SR , Sremanakova J , Lapitan MCM , Qureshi AU , Drake TM , et al. Malnutrition and nutritional screening in patients undergoing surgery in low and middle income countries: A systematic review. JCSM Clin Rep 2022;7:79–92.

[10] Correia MI , Davisson T . Nutrition Screening vs Nutrition Assessment: What's the Difference? Nutr Clin Pract 2018;33:62–72. 28727954 10.1177/0884533617719669

[11] Fried LP , Tangen CM , Walston J , Newman AB , Hirsch C , Gottdiener J , et al. Frailty in older adults: evidence for a phenotype. J Gerontol A Biol Sci Med Sci 2001;56:M146–M157. 11253156 10.1093/gerona/56.3.m146

[12] Norman K , Pichard C , Lochs H , Pirlich M . Prognostic impact of disease‐related malnutrition. Clin Nutr 2008;27:5–15. 18061312 10.1016/j.clnu.2007.10.007

[13] GlobalSurg Collaborative and NIHR Global Health Unit on Global Surgery . The impact of malnutrition on early outcomes after cancer surgery: an international, prospective cohort study. Lancet Global 2023;11:E341–E349. 10.1016/S2214-109X(22)00550-2 36796981

[14] Sulo S , Schwander B , Brunton C , Gomez G , Misas JD , Gracia DA , et al. Nutrition‐focused care for community‐living adults: healthcare utilization and economic benefits. Value Health Reg Issues 2022;32:70–77. 36099802 10.1016/j.vhri.2022.08.005

[15] Burden ST , Gibson DJ , Lal S , Hill J , Pilling M , Soop M , et al. Pre‐operative oral nutritional supplementation with dietary advice versus dietary advice alone in weight‐losing patients with colorectal cancer: single‐blind randomized controlled trial. J Cachexia Sarcopenia Muscle 2017;8:437–446. 28052576 10.1002/jcsm.12170 PMC5476846

[16] Baldwin C , de van der Schueren MAE , Kruizenga HM , Weekes CE Dietary advice with or without oral nutritional supplements for disease‐related malnutrition in adults. Cochrane Database Syst Rev 2021;2021:CD002008. 10.1002/14651858.CD002008.pub5 PMC8691169 34931696

[17] Cawood AL , Burden ST , Smith T , Stratton RJ . A systematic review and meta‐analysis of the effects of community use of oral nutritional supplements on clinical outcomes. Ageing Res Rev 2023;88:101953. 37182743 10.1016/j.arr.2023.101953

[18] Söderström L , Rosenblad A , Thors Adolfsson E , Bergkvist L . Malnutrition is associated with increased mortality in older adults regardless of the cause of death. Br J Nutr 2017;117:532–540. 28290264 10.1017/S0007114517000435

[19] Dent E , Hoogendijk E , Visvanathan R , Wright O . Malnutrition screening and assessment in hospitalised older people: a review. J Nutr Health Aging 2019;23:431–441.31021360 10.1007/s12603-019-1176-z

[20] Nyberg ST , Singh‐Manoux A , Pentti J , Madsen IEH , Sabia S , Alfredsson L , et al. Association of healthy lifestyle with years lived without major chronic diseases. JAMA Intern Med 2020;180:760–768. 32250383 10.1001/jamainternmed.2020.0618 PMC7136858

[21] Norman K , Haß U , Pirlich M . Malnutrition in older adults—recent advances and remaining challenges. Nutrients 2021;13:2764. 34444924 10.3390/nu13082764 PMC8399049

[22] Almohaisen N , Gittins M , Todd C , Sremanakova J , Sowerbutts AM , Aldossari A , et al. Prevalence of undernutrition, frailty and sarcopenia in community‐dwelling people aged 50 years and above: systematic review and meta‐analysis. Nutrients 2022;14:1537. 35458101 10.3390/nu14081537 PMC9032775

[23] Cereda E , Pedrolli C , Klersy C , Bonardi C , Quarleri L , Cappello S , et al. Nutritional status in older persons according to healthcare setting: A systematic review and meta‐analysis of prevalence data using MNA®. Clin Nutr 2016;35:1282–1290. 27086194 10.1016/j.clnu.2016.03.008

[24] Almohaisen N , Gittins M , Todd C , Burden S . Estimating the prevelance of older people at risk of undernutrition, frailty and sarcopenia using UK Biobank standardised population level data. Clin Nutr ESPEN 2023;54:723.

[25] Laur CV , McNicholl T , Valaitis R , Keller HH . Malnutrition or frailty? Overlap and evidence gaps in the diagnosis and treatment of frailty and malnutrition. Appl Physiol Nutr Metab 2017;42:449–458. 28322060 10.1139/apnm-2016-0652

[26] Jones DJ , Baldwin C , Lal S , Stanmore E , Farrer K , Connolly E , et al. Priority setting for adult malnutrition and nutritional screening in healthcare: a James Lind Alliance. J Hum Nutr Diet 2020;33:274–283. 31858685 10.1111/jhn.12722

[27] Sudlow C , Gallacher J , Allen N , Beral V , Burton P , Danesh J , et al. UK Biobank: an open access resource for identifying the causes of a wide range of complex diseases of middle and old age. PLoS Med 2015;12:e1001779. 25826379 10.1371/journal.pmed.1001779 PMC4380465

[28] Fry A , Littlejohns TJ , Sudlow C , Doherty N , Adamska L , Sprosen T , et al. Comparison of sociodemographic and health‐related characteristics of UK Biobank participants with those of the general population. Am J Epidemiol 2017;186:1026–1034. 28641372 10.1093/aje/kwx246 PMC5860371

[29] Elia M , Stratton R . Geographical inequalities in nutrient status and risk of malnutrition among English people aged 65 y and older. Nutrition 2005;21:1100–1106. 16308132 10.1016/j.nut.2005.03.005

[30] Jin M , Du H , Zhang Y , Zhu H , Xu K , Yuan X , et al. Characteristics and reference values of fat mass index and fat free mass index by bioelectrical impedance analysis in an adult population. Clin Nutr 2019;38:2325–2332. 30389251 10.1016/j.clnu.2018.10.010

[31] AlMohaisen N , Gittins M , Todd C , Burden S . What is the overlap between malnutrition, frailty and sarcopenia in the older population? Study protocol for cross‐sectional study using UK Biobank. PLoS ONE 2022;17:e0278371. 36472992 10.1371/journal.pone.0278371 PMC9725160

[32] Yeung SSY , Chan RSM , Kwok T , Lee JSW , Woo J . Malnutrition according to GLIM criteria and adverse outcomes in community‐dwelling chinese older adults: a prospective analysis. J Am Med Dir Assoc 2021;22:1953–9.e4. 33153909 10.1016/j.jamda.2020.09.029

[33] Petermann‐Rocha F , Pell JP , Celis‐Morales C , Ho FK . Frailty, sarcopenia, cachexia and malnutrition as comorbid conditions and their associations with mortality: a prospective study from UK Biobank. J Public Health (Oxf) 2022;44:e172–e180. 33423060 10.1093/pubmed/fdaa226 PMC9234318

[34] Prokopidis K , Chambers E , Ni Lochlainn M , Witard OC . Mechanisms linking the gut‐muscle axis with muscle protein metabolism and anabolic resistance: implications for older adults at risk of sarcopenia. Front Physiol 2021;12:770455. 34764887 10.3389/fphys.2021.770455 PMC8576575

[35] Calcaterra L , Abellan van Kan G , Steinmeyer Z , Angioni D , Proietti M , Sourdet S . Sarcopenia and poor nutritional status in older adults. Clin Nutr 2024;43:701–707. 38320461 10.1016/j.clnu.2024.01.028

[36] Nicholson BD , Aveyard P , Bankhead CR , Hamilton W , Hobbs FDR , Lay‐Flurrie S . Determinants and extent of weight recording in UK primary care: an analysis of 5 million adults' electronic health records from 2000 to 2017. BMC Med 2019;17:222. 31783757 10.1186/s12916-019-1446-y PMC6883613

[37] Mills CM , Keller HH , DePaul VG , Donnelly C . Factors associated with the development of high nutrition risk: data from the Canadian Longitudinal Study on Aging. Can J Aging/La Revue canadienne du vieillissement 2024;43:153–166. 10.1017/S0714980823000545 37749058

[38] Sanchez‐Rodriguez D , Locquet M , Reginster J‐Y , Cavalier E , Bruyère O , Beaudart C . Mortality in malnourished older adults diagnosed by ESPEN and GLIM criteria in the SarcoPhAge study. J Cachexia Sarcopenia Muscle 2020;11:1200–1211. 32657045 10.1002/jcsm.12574 PMC7567139

[39] Miller LMS , Tancredi DJ , Kaiser LL , Tseng JT . Midlife vulnerability and food insecurity: Findings from low‐income adults in the US National Health Interview Survey. PLoS ONE 2020;15:e0233029. 32658927 10.1371/journal.pone.0233029 PMC7357765

[40] Dickinson A , Godfrey‐Smythe A , Halliday SV , Ikioda F , Kapetanaki AB , Wills W . Food security and food practices in later life: a new model of vulnerability. Ageing Soc 2022;42:2180–2205.

