# Supplementary material for: Overall mortality for community‐dwelling adults over 50 years at risk of malnutrition
Source: J Cachexia Sarcopenia Muscle. 2024 Aug 29;15(6):2509–18. doi: 10.1002/jcsm.13585 (PMC11634492; doi:10.1002/jcsm.13585)
Supplement: Supplementary file 1 — Data S1. Kaplan Meier plot comparing MUST definition of malnutrition defining at risk vs not for males and female separately. Data S2. Years of Life lost (per 100,000 persons) for those with malnutrition by age at death and gender. Data S3. Years of Life lost (per 100,000 persons) for those with malnutrition by age at death and gender but did not have cancer at baseline. Data S4. Sensitivity Analysis Hazard ratios and 95% confidence intervals for survival analysis (with shared frailty) comparing those at risk of malnutrition vs low risk of malnutrition. [file JCSM-15-2509-s001.docx]

**Supplementary Material**

**Supplementary Figure S1: Kaplan Meier plot comparing MUST definition of malnutrition defining at risk vs not for males and female separately.**

**Supplementary Figure S2: Years of Life lost (per 100,000 persons) for those with malnutrition by age at death and gender**

M=male F=female

**Supplementary Figure S3: Years of Life lost (per 100,000 persons) for those with malnutrition by age at death and gender but did not have cancer at baseline**

M=male F=female

**Supplementary Table S1: Sensitivity Analysis Hazard ratios and 95% confidence intervals for survival analysis (with shared frailty) comparing those at risk of malnutrition vs low risk of malnutrition**

| **Sensitivity Analysis - Shared Frailty model** |  | **95% C.I.** | |
| --- | --- | --- | --- |
| **Malnutrition (MUST)** | **Hazard Ratio** | **Lower** | **Upper** |
| **No Adjustment (Mod 1)** |  |  |  |
| Low Risk |  |  |  |
| At Risk of Malnutrition | 1.30 | 1.26 | 1.34 |
| **Adjusted for baseline confounders (Mod 2)** |  |  |  |
| Low Risk |  |  |  |
| At Risk of Malnutrition | 1.15 | 1.11 | 1.18 |
| **Adjusted for time-varying cancer presence (Mod 3)** |  |  |  |
| Low Risk |  |  |  |
| At Risk of Malnutrition | 1.15 | 1.11 | 1.19 |
| **Interaction model with Gender** |  |  |  |
| Low Risk |  |  |  |
| At Risk of Malnutrition | 1.14 | 1.09 | 1.19 |
| Female |  |  |  |
| Male | 1.50 | 1.45 | 1.55 |
| Health Status*Gender Interaction | 1.02 | 0.96 | 1.09 |
| **Restricted to those without cancer at baseline (Mod 4) only** |  |  |  |
| Low Risk |  |  |  |
| At Risk of Malnutrition | 1.13 | 1.09 | 1.17 |

Mod1 = No adjustment,

Mod 2 = adjust for known fixed confounders: gender, ethnicity, qualification, and deprivation (in the form of the Townsend Score), and co-morbidities hypertension, diabetes, ischaemic heart disease, osteoporosis, Parkinson’s, thyroid disease, cerebrovascular disease, chronic kidney disease, atrial fibrillation, anaemia, and respiratory disease, smoking status, baseline cancer presence.

Mod 3 = plus adjustment for time-varying cancer occurrence

Mod 4 = restricted sample of healthy participants without cancer

**Supplementary references**

S1. World Health Organisation. UN Decade of Healthy Ageing: Plan of Action 2021-2030. h<ttps://cdn.who.int/media/docs/default-source/decade-of-healthy-ageing/decade.> 2023. Accessed 01/07/24.

S2. Office for Health Improvement and Disparities. A Consensus on Healthy Ageing: Policy paper h<ttps://www.gov.uk/government/publications/healthy-ageing-consensus-statement/a-consensus-on-healthy-ageing.> 2023. h<ttps://www.gov.uk/government/publications/healthy-ageing-consensus-statement/a-consensus-on-healthy-ageing.> Accessed 2/3/24.

S3. Office for Health Improvement and Disparities. A Consensus on Healthy Ageing: Policy paper https://www.gov.uk/government/publications/healthy-ageing-consensus-statement/a-consensus-on-healthy-ageing. 2023. [https://www.gov.uk/government/publications/healthy-ageing-consensus-statement/a-consensus-on-healthy-ageing. Accessed 2/3/24](https://www.gov.uk/government/publications/healthy-ageing-consensus-statement/a-consensus-on-healthy-ageing.%20Accessed%202/3/24).
